# Identification of biochemical and cytotoxic markers in cocaine treated PC12 cells

**DOI:** 10.1038/s41598-018-21182-7

**Published:** 2018-02-09

**Authors:** Ramesh B. Badisa, Chyree S. Batton, Elizabeth Mazzio, Samuel C. Grant, Carl B. Goodman

**Affiliations:** 1 College of Pharmacy and Pharmaceutical Sciences, Florida A&M University, Tallahassee, FL 32307 USA; 2 The National High Magnetic Field Laboratory, Florida State University, Tallahassee, FL 32310 USA

## Abstract

Cocaine is one of the powerful addictive drugs, widely abused in most Western countries. Because of high lipophilic nature, cocaine easily reaches various domains of the central nervous system (CNS) and triggers different levels of cellular toxicity. The aim of this investigation was to reproduce cocaine toxicity in differentiated PC12 cells through quantitative knowledge on biochemical and cytotoxicity markers. We differentiated the cells with 0.1 μg/ml nerve growth factor (NGF) for 5 days, followed by treatment with cocaine for 48 h at *in vivo* and *in vitro* concentrations. Results indicated that cocaine at *in vivo* concentrations neither killed the cells nor altered the morphology, but decreased the mitochondrial membrane potential that paralleled with increased lactate and glutathione (GSH) levels. On the other hand, cocaine at *in vitro* concentrations damaged the neurites and caused cell death, which corresponded with increased reactive oxygen species (ROS) generation, plasma membrane damage, and GSH depletion with no detectable nitric oxide (NO) level. While direct understanding of cocaine and cell interaction under *in vivo* animal models is impeded due to high complexity, our present *in vitro* results assisted in understanding the onset of some key events of neurodegenerative diseases in cocaine treated neuronal cells.

## Introduction

Neuronal development, which involves generation, migration, and differentiation of neurons, is essential for a complete and functional nervous system. Similarly, neuronal outgrowth^1^, branching^2^, and retraction^3^ play an important role in neuronal networking process at embryonic and adult stages. Injury to such neurons by external insults can affect neuronal structure and networking system, and at times can cause immediate neuronal death^4^. Studies showed that certain external insults like substances of drug abuse can induce changes in the structural integrity of neurons and damage their networking processes^5–7^.

Cocaine is one of the widely abused drugs that cause psycho-stimulatory effects in the central nervous system (CNS). It elevates the mood of addicts initially but leads to severe psychological disorders^8^, -such as depression^9^, anger, aggressiveness, and paranoia^10^ due to imbalance of neurotransmitters. Irrespective of route of intake, cocaine consumption causes severe negative effects in the body such as increased blood pressure and cytotoxicity in all vital organs of the body like heart and kidneys^11–13^. In the CNS, cocaine was shown to induce death of dopaminergic neurons^14^. Owing to its lipophilic and hydrophilic nature, cocaine easily crosses placenta^15^, thus its use by pregnant women could lead to various complications during fetal development^16^ or induce abortion^17^, or result in premature labor.

Very few reports are available for the quantification of cocaine effects on neurite outgrowth, morphological changes or neuronal loss under *in vitro* conditions. In this study, we investigated whether cocaine-induced changes on the structural integrity of neurons and neurites observed *in vivo* could be reproduced in cell cultures for better understanding and quantification of those changes. In addition, we also evaluated several biochemical changes both at *in vivo* pharmacological (low) and *in vitro* concentrations (high) of cocaine. At pharmacological concentrations, dopamine (DA) level, general mitochondrial activity, membrane potential, lactate release, and glutathione (GSH) level were measured (Biochemical markers), while at *in vitro* concentrations, we measured cytotoxicity markers such as production of reactive oxygen species (ROS), and lactate dehydrogenase (LDH) release, GSH level, and nitric oxide (NO) generation. We employed rat pheochromocytoma PC12 cells as a model culture in this study.

## Results

### Differentiation

**Figure 1:**
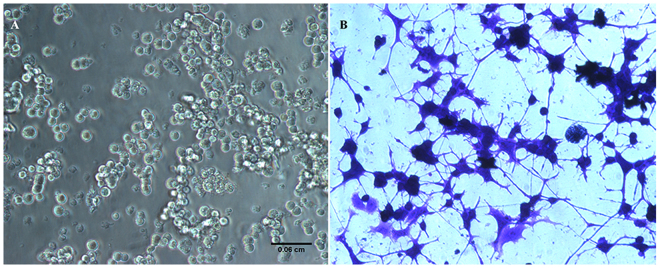
Morphological features of PC12 cells. Undifferentiated cells grow as round aggregates (**A**) in the medium (unstained). NGF exposed cells (**B**) developed extensive growth of neurites (stained with crystal violet dye) with intercellular junctions. Cells were photographed under an inverted phase contrast microscope with 20x objective. Scale bar: 0.06 mm.

PC12 cells usually grow as floating aggregates in culture medium. In our study, undifferentiated PC12 cells appeared oval to round shape (Fig. 1A). When exposed to NGF at 0.1 μg/ml for five days, the post mitotic cells attached to the collagen coated plates and showed the de-nova neurite outgrowth. Profusely differentiated cells exhibited clear signs of bi or tri polar neurites which appeared slender, mostly straight but branched at some areas with sharp edges (Fig. 1B). In addition, the neurites formed intercellular connections at several areas, demonstrating one of the important features meant for communication by the differentiated neurons. The cell-body mostly appeared as polygonal.

### Neuronal characteristics

**Figure 2:**
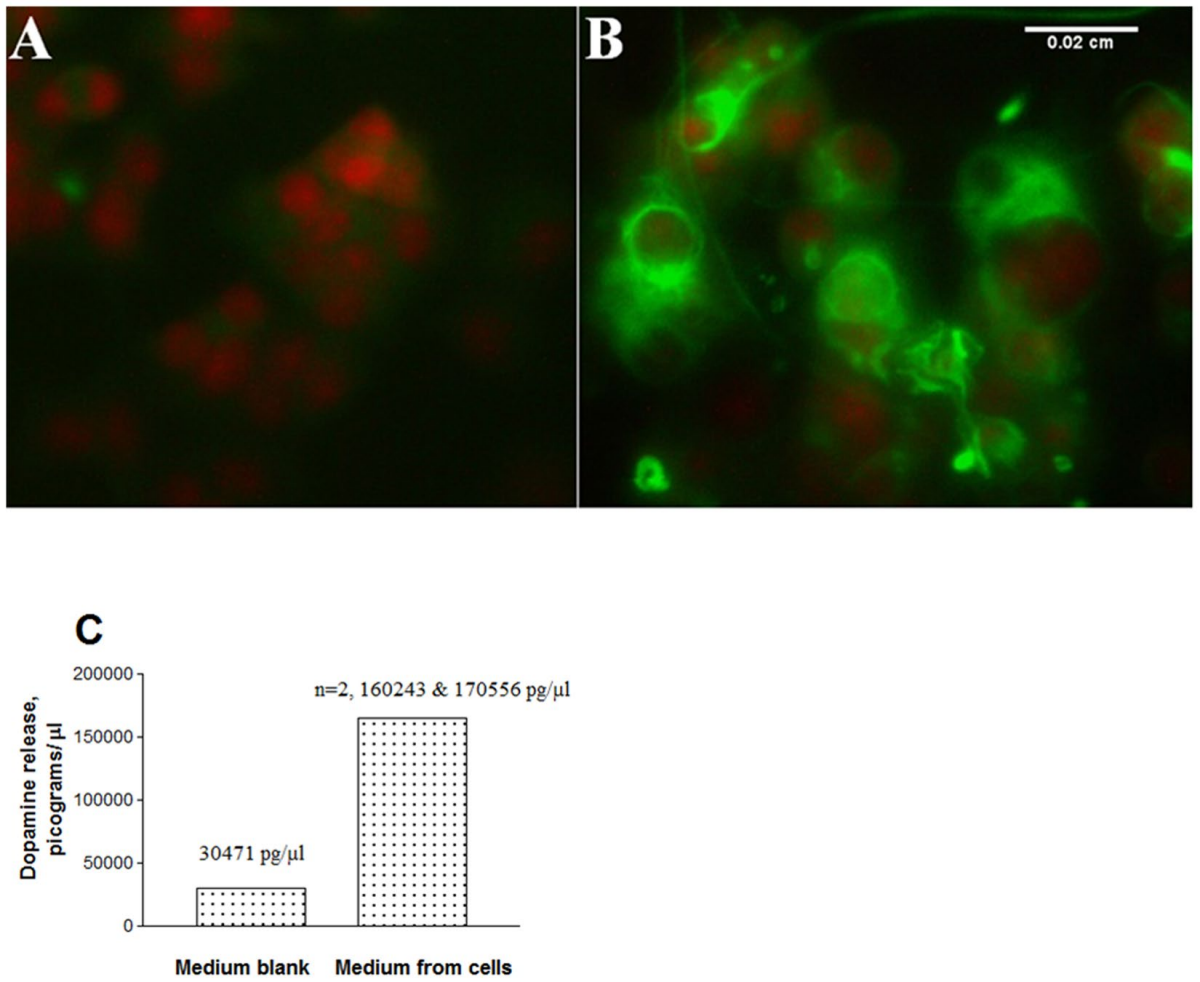
Neuronal-like characteristics of differentiated PC12 cells. After fixation and permeabilization, the undifferentiated (**A**) or NGF exposed (**B**) cells in 96-well plates were stained with primary rabbit anti-rat NF 200 kD and secondary goat anti-rabbit Alexa Fluor® 488. Cells were counterstained for nuclei with PI and photomicrographed using an inverted fluorescent microscope with a 25x objective. Scale bar: 0.02 mm. For dopamine analysis (**C**), the medium (50 μl) from differentiated cells was mixed with 100 μl 0.1 N PCA and analyzed by reverse phase HPLC C-18 ODS column at an isocratic flow rate of 1 ml/min.

It is well known that differentiated PC12 cells exhibit neuronal phenotype^18^. To confirm the neuronal characteristics under our experimental conditions, we stained both undifferentiated and differentiated cells for the presence of neurofilaments, which are the intermediate filaments found only in neurons. The red structures in propidium iodide (PI) stained cells represent the nuclei. While lack of staining in undifferentiated cell indicated the absence of neurofilaments (Fig. 2A), the presence of significant staining (green) in NGF-differentiated cells clearly showed the neurofilaments (Fig. 2B).

Differentiated PC12 cells behave as dopaminergic neurons^18^ and release detectable amount of DA in culture medium. When we analyzed for DA from the medium of differentiated cells by HPLC, it was found that cells released a high level of DA (Fig. 2C). For instance, the amount of DA in the medium blank was 30471 pg/μl, while the medium from differentiated cells contained an average of 165399.5 ± 5156.5 pg/μl DA, -an increase of 5.4 folds. Based on the presence of neurofilaments, neurite outgrowth and DA release, it is clear that the differentiated PC12 cells exhibited neuronal characteristics.

### Effect of cocaine at *in vivo* pharmacological doses on different cellular parameters

**Figure 3:**
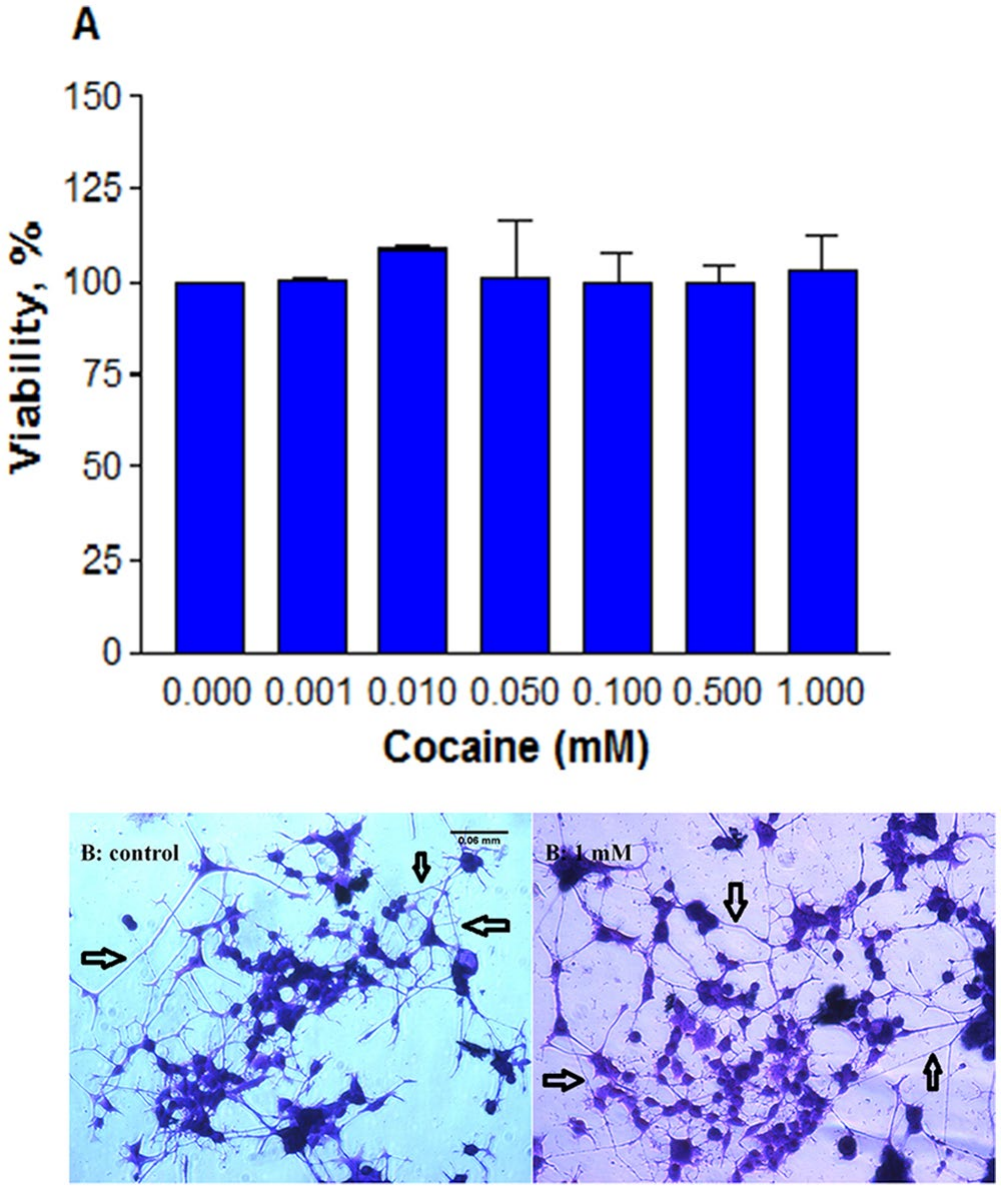
Effect of pharmacological doses of cocaine on viability and morphology. Differentiated cells were treated with PBS (control) or cocaine for 48 h in 96-well plates. Crystal violet stained cells (*n* = 4, P = 0.99) were assessed for viability (**A**); morphology of control and 1 mM cocaine treated cells was photographed under an inverted phase contrast 1X-70 Olympus microscope with 20x objective (**B**), showing neurite connections with black arrows. Data are expressed as mean ± SEM, insignificant compared to the PBS control. Scale bar 0.06 cm.

Cocaine concentrations in brain cells of drug addicts ranged from nano- to micromolar^19^. In view of this, we initially treated the differentiated cells with cocaine at various lower concentrations (0.001, 0.01, 0.05, 0.1, 0.5 and 1 mM final) for 48 h to simulate a wide range of *in vivo* pharmacological doses and evaluated for cell viability. The data clearly indicated that cocaine did not induce significant (*n* = 4, P = 0.99) cell death at any of these doses compared to the control (Fig. 3A). Similarly, the cell morphology and the extent of neurite branching and connections appeared the same both in the control and at 1 mM cocaine (Fig. 3B). Black arrows point the interneurite connections.

**Figure 4:**
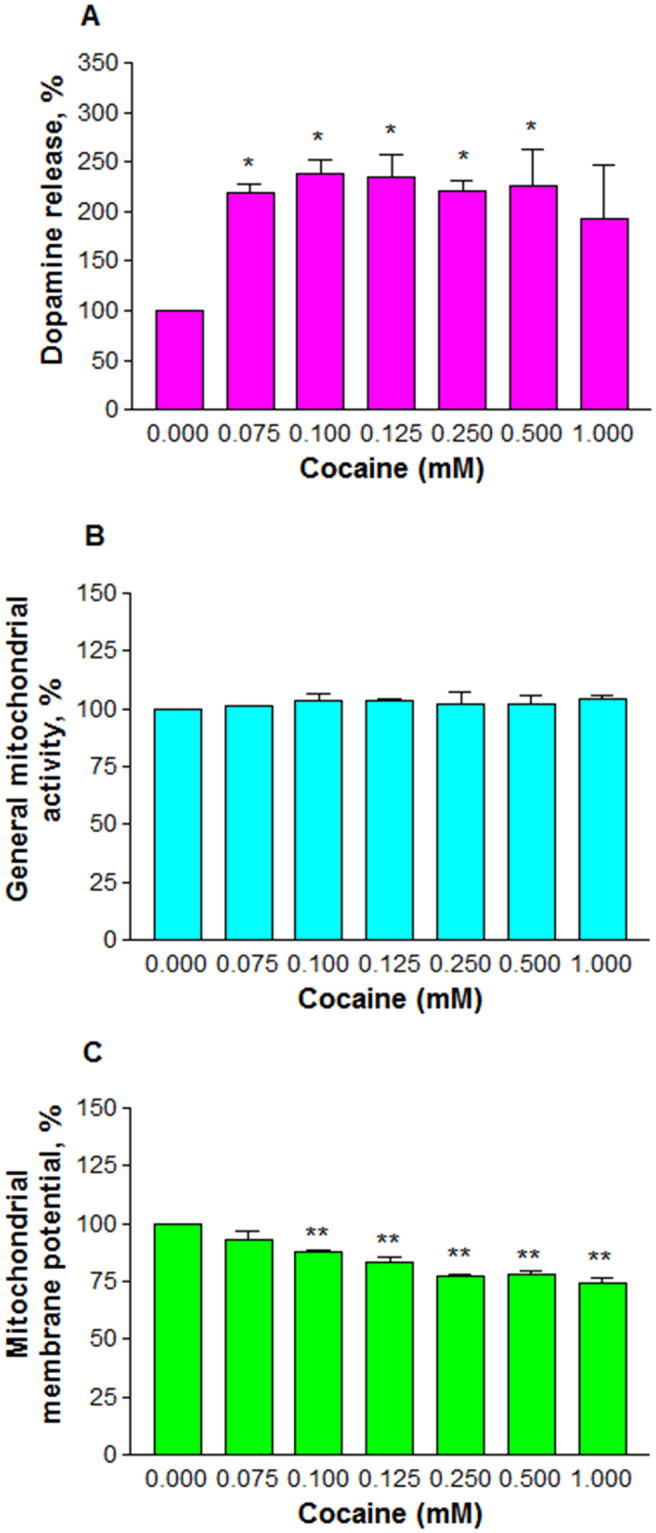
Effect of pharmacological doses of cocaine on cell biochemistry. Differentiated cells were treated with PBS (control) or cocaine for 48 h in 96-well plates. The medium (50 μl) was mixed with 100 μl 0.1 N PCA and analyzed for dopamine release (*n* = 3, *P = 0.03) by HPLC (**A**) or the cells were mixed with 10 μl of MTS (*n* = 3, P = 0.87) 1 h prior to the end-point for general mitochondrial activity (**B**) or stained with Rh 123 (*n* = 8, **P = 0.0001) for mitochondrial membrane potential (**C**). Data are expressed as mean ± SEM, significant compared to the PBS controls.

Since differentiated PC12 cells behaved like dopaminergic neurons, we then studied the effect of cocaine on DA level. The cells were treated with cocaine at 0.075, 0.1, 0.125, 0.25, 0.5 and 1 mM for 48 h. Data obtained by HPLC analysis indicated that the amount of DA at any cocaine treatment was significantly (*n* = 3, P = 0.03) higher than the control cells (Fig. 4A). There was a dose-dependent increase in DA up to 0.25 mM cocaine, and thereafter a decreasing trend was observed. Compared to the control (100%), the average release in DA was (±SEM) 218.3 ± 8.3, 238 ± 15.1, 234.5 ± 19.1, 221.3 ± 10.2, 226.6 ± 35.9, and 192 ± 55.1% at 0.075, 0.1, 0.125, 0.25, 0.5 and 1 mM cocaine respectively.

Next, we evaluated the role of cocaine on the general mitochondrial activity. For this purpose, the cells were treated with cocaine at 0.075, 0.1, 0.125, 0.25, 0.5 and 1 mM for 48 h. The results clearly indicated that cocaine treatment did not interfere (*n* = 3, P = 0.87) in the general mitochondrial activity of the cells (Fig. 4B). We then measured the mitochondrial membrane potential using the fluorescence probe Rh 123 (10 μM). This lipophilic dye permeates mitochondria easily and interacts with the inner membrane at micro molar concentrations. The accumulation in mitochondria is proportional to membrane potential. The data clearly indicated that cocaine treatment significantly decreased (*n* = 8, P = 0.0001) the mitochondrial membrane potential in the cells (Fig. 4C). Compared to the control (100%), the average decrease of the membrane potential was (±SEM) 92.9 ± 3.5, 88 ± 0.6, 83.3 ± 1.9, 77.2 ± 1.1, 77.9 ± 1.7, and 74.7 ± 1.9% at 0.075, 0.1, 0.125, 0.25, 0.5 and 1 mM cocaine respectively.

**Figure 5:**
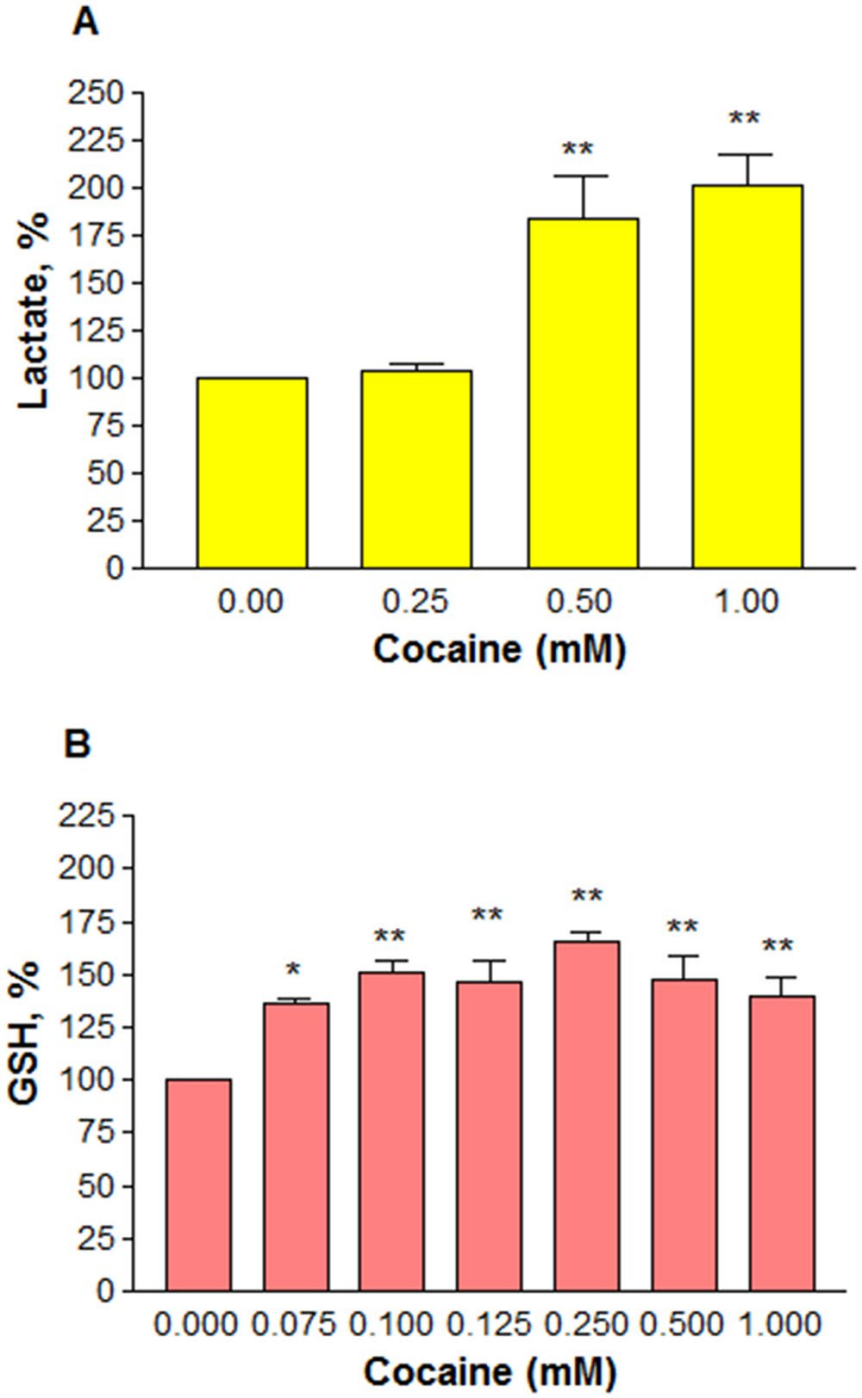
Effect of pharmacological doses of cocaine on the levels of lactate (**A**) or GSH (**B**). Differentiated cells in phenol-red free medium were treated with PBS (control) or cocaine for 48 h. Kit reagent (20 μl/well) was added for lactate measurement (*n* = 8, **P = 0.001), or fixed cells were evaluated for GSH level (*n* = 3, *P = 0.041 or **P = 0.013). Data are expressed as mean ± SEM, significant compared to the PBS controls.

Disruption in mitochondrial potential usually switches the cells to anaerobic glycolysis for survival. Under such condition, pyruvate is oxidized to lactate and released into the medium. In order to study whether loss in membrane potential (Fig. 4C) resulted in the release of lactate, we treated the cells with cocaine at 0.25, 0.5 and 1 mM for 48 h. It was observed that cocaine treatment caused a significant (*n* = 8, P = 0.001) release of lactate compared to the control cells (Fig. 5A**)**. No significant release of lactate was detected with cocaine treatments at 0.25 mM or lower. Compared to the control (100%), the average release of lactate was (±SEM) 104 ± 3.2, 184.5 ± 20.1 and 201.3 ± 14.9% at 0.25, 0.5 and 1 mM cocaine respectively. Under this situation, we sought to know the role of cocaine on GSH level. The cells were treated with 0.075, 0.1, 0.125, 0.25, 0.5 and 1 mM cocaine for 48 h. There was a dose-dependent significant (*n* = 3, P = 0.041 to 0.013) increase in GSH level at all treatments compared to the control (Fig. 5B). Compared to the control (100%), the average increase of GSH in the cells was ( ± SEM) 135.8 ± 2.4, 150.5 ± 6.5, 146 ± 9.9, 165.2 ± 4.9, 147.1 ± 12 and 139.9 ± 8.3% at 0.075, 0.1, 0.125, 0.25, 0.5 and 1 mM cocaine respectively.

### Effect of cocaine at *in vitro* doses

Because there was no severe cytotoxic effect of cocaine on cells at different pharmacological concentrations, cocaine doses in the subsequent studies were increased. Based on several *in vitro* studies^20–23^, we tested the cells at 2, 3 and 4 mM cocaine for 48 h and evaluated for several cellular parameters as shown below:

#### Cell viability, ROS and membrane integrity

**Figure 6:**
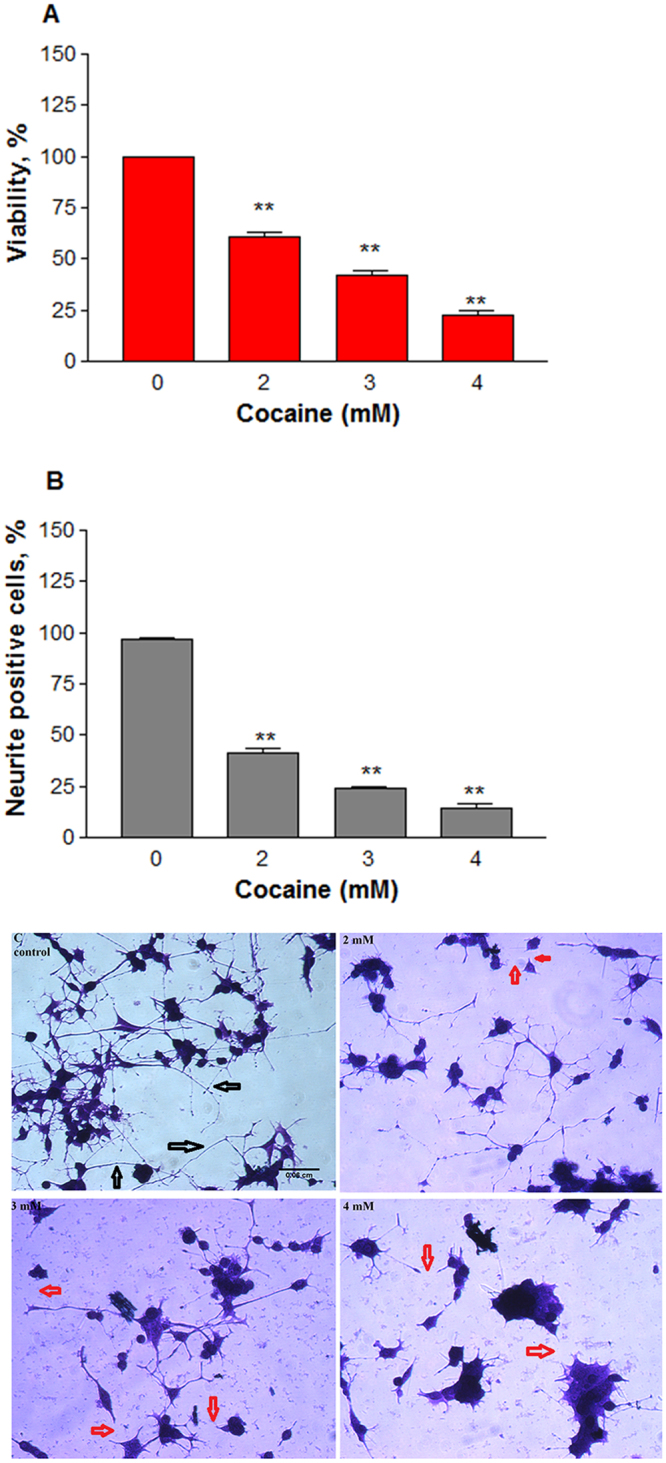
Cytotoxic effect of cocaine at higher doses. Differentiated cells in phenol-red free medium were treated with PBS (control) or cocaine for 48 h. Cell viability (**A**) was assessed (*n* = 12, **P = 0.001) by the crystal violet dye uptake method; neuronal extensions greater than 2-body diameter in length of 100 cells per well at four randomly chosen field areas were measured (*n* = 8, **P = 0.001) and quantified (**B**); the crystal violet stained cells were photographed under an inverted phase contrast 1X-70 Olympus microscope with 20x objective (**C**), showing neurite connections with black arrows or disconnected neurites upon cocaine treatments with red arrows. Data are expressed as mean ± SEM, significant compared to the PBS controls. Scale bar 0.06 cm.

Higher doses of cocaine caused a significant (*n* = 12, P = 0.001) dose-dependent decrease in the cell viability (Fig. 6A). For instance, compared to the control (100%), the average decrease in the viability (±SEM) at 2, 3, and 4 mM cocaine was 61.2 ± 1.9, 41.8 ± 2.7, and 22.8 ± 1.8, respectively. The cocaine lethal concentration, LC_50_, where 50% cells were killed, was found to be 2.605 mM. Neurite elongation was also decreased significantly with increasing cocaine concentrations. For instance, quantification of cellular extensions greater than 2-body diameter in length^24^ confirmed that cocaine treatment indeed caused a significant (*n* = 8, P = 0.001) dose-dependant decrease in neurite length (Fig. 6B), shown in red arrows (Fig. 6C). Compared to the control (100%), the average neurite outgrowth (±SEM) at 2, 3 and 4 mM cocaine was 41.3 ± 1.9, 23.8 ± 1.1 and 14.3 ± 1.9%, respectively.

**Figure 7:**
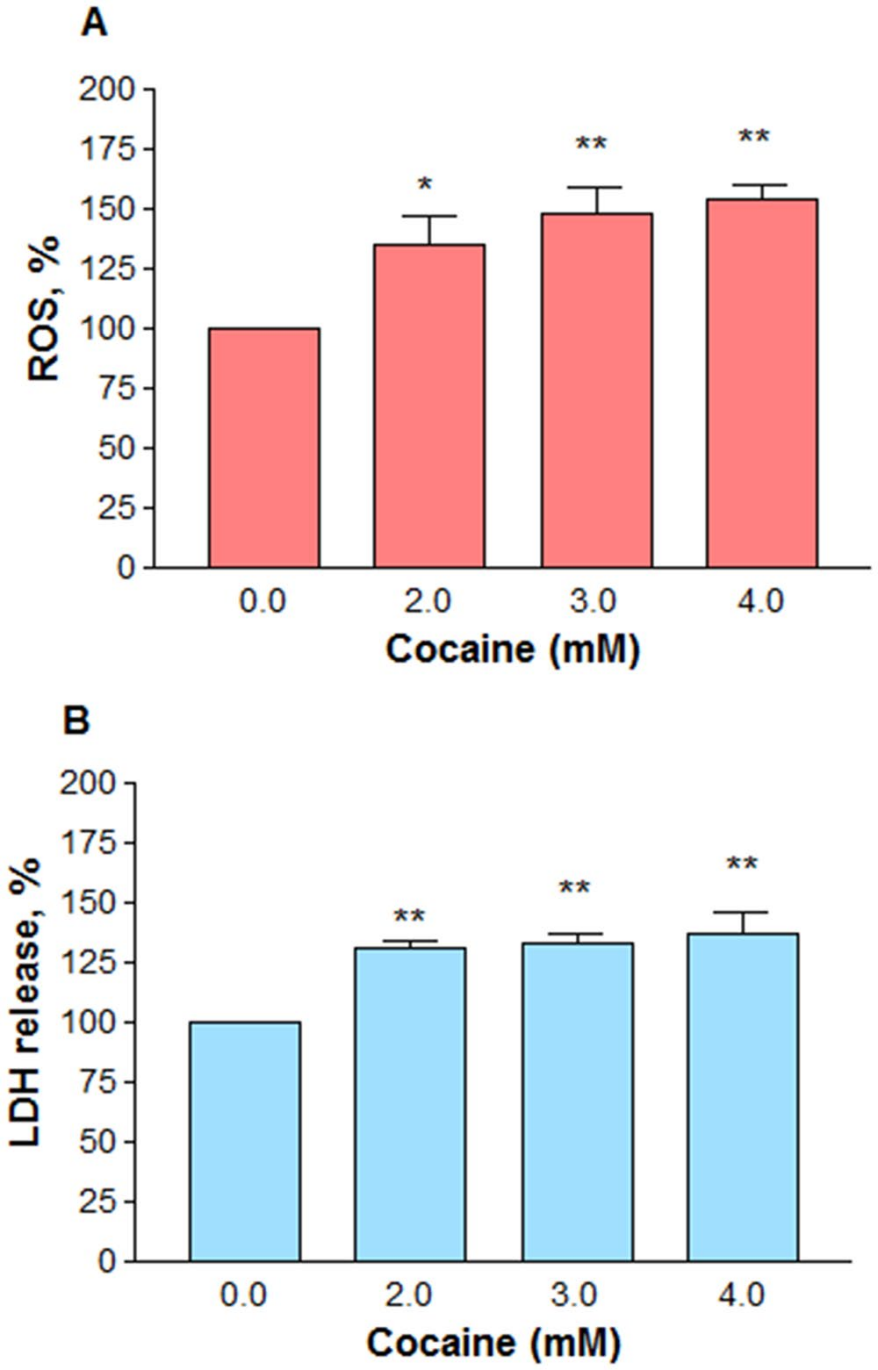
Effect of higher doses of cocaine on ROS generation and membrane integrity. Differentiated cells in phenol-red free medium were treated with PBS (control) or cocaine for 48 h. ROS generation in the cells loaded with DCFDA probe (10 μM) was measured (*n* = 12, **P = 0.0003) with the excitation and emission filters set at 485 nm and 530 nm respectively in a fluorescence micro plate reader (**A**); membrane integrity was determined by measuring LDH release (**B**) from the cells (*n* = 10, **P = 0.001) at 490 nm in a micro plate reader. Data are expressed as mean ± SEM, significant compared to the PBS controls.

We speculated that ROS could be the reason for decrease in the cell viability due to its interaction with mitochondria. So, after treating the cells with cocaine at 2, 3 and 4 mM for 48 h, the ROS level was measured by staining the cells with the fluorescence probe 2′,7′ –dichloro fluorescin diacetate. It was found that there was a significant (*n* = 12, P = 0.0003) dose-dependent increase in ROS level compared to the control cells (Fig. 7A). The average increase in ROS at 2, 3 and 4 mM cocaine was (±SEM) 135.2 ± 12.1, 148.2 ± 11.3 and 154.2 ± 5.3%, respectively compared to the control (100%). Since high level of ROS can compromise the cell membrane integrity, we then measured the release of cytosolic LDH into the media. A dose-dependent significant (*n* = 10, P = 0.001) increase in LDH was observed (Fig. 7B). For instance, the average LDH increase at 2, 3, and 4 mM cocaine was (±SEM) 130.7 ± 3.1, 133.4 ± 3.7 and 137.3 ± 8.2%, respectively compared to the control.

#### Decreased GSH level

**Figure 8:**
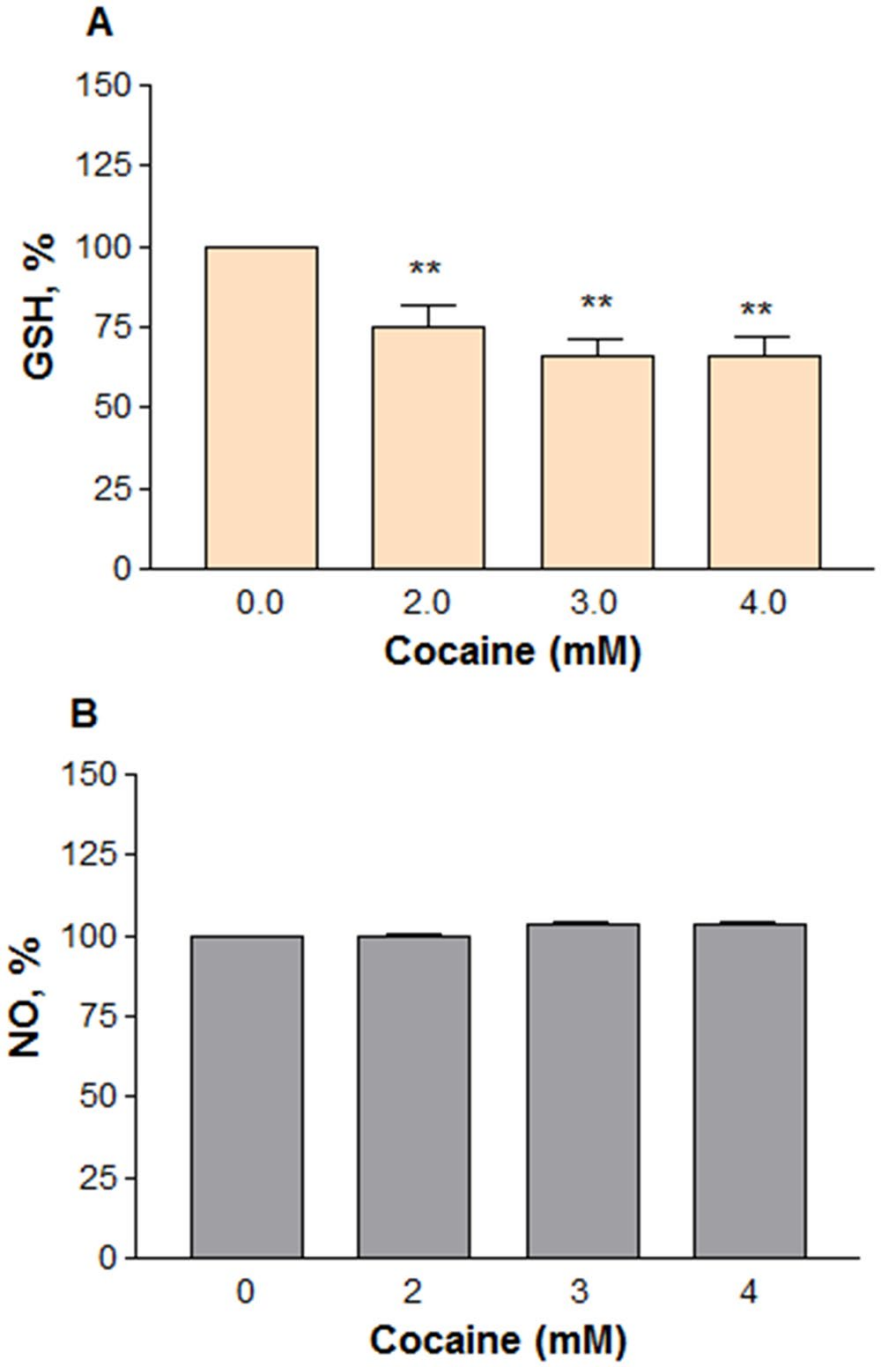
Effect of higher doses of cocaine on GSH or NO level. Differentiated cells in phenol-red free medium were treated with PBS (control) or cocaine for 48 h. GSH (**A**) level in fixed cells (*n* = 7, **P = 0.0004) was measured as per established procedure at 412 nm in a micro plate reader; for NO detection (**B**), 100 μl of medium was transferred into a new 96-well plate (*n* = 9, P = 0.6) and mixed with equal volume of Griess reagent, followed by 10 min incubation in the dark. The absorbance readings at 540 nm were measured in a micro plate reader. Data are expressed as mean ± SEM and compared to the PBS controls.

Glutathione is one of the most available antioxidant substances in the cells. In order to explore the potential role of high cocaine concentrations on total glutathione levels, the PC12 cells were treated with different cocaine concentrations (2–4 mM) for 48 h. It was found that cocaine treatment caused a significant (*n* = 7, P = 0.0004) decrease in GSH level compared to the control (Fig. 8A). The decrease in GSH levels was (±SEM) 25 ± 7.1, 34.1 ± 5.4 and 33.9 ± 5.8% of the control value (100%) at 2, 3 and 4 mM cocaine, respectively.

#### Lack of inflammatory response

Overproduction of nitric oxide indicates one of the inflammatory responses. While nitric oxide (NO) is unstable at physiological pH, its interaction with air eventually leads in the formation of a more stable nitrite that can be detected easily by Greiss reagent. In order to understand the role of cocaine on inflammation, we treated the cells with cocaine at 2, 3 and 4 mM doses for 48 h. The cells were not challenged with bacterial lipopolysaccharide or γ interferon during the treatment period because differentiated PC12 cells have nNOS^25,26^, and we wanted to know if cocaine induces this enzyme for excessive NO production. The data indicated clearly that in comparison to the control, cocaine at any concentration did not stimulate or inhibit NO production significantly in the cells (*n* = 9, P = 0.6,) after 48 h (Fig. 8B).

## Discussion

Neurons are the basic components of CNS regarding signal transmission needed for cell-cell communication through dendrites and axons. Under *in vitro* condition, PC12 cells were routinely employed as a neuronal model to study neurites^27–29^. These cells were also used extensively as a model to study cocaine effects^30–32^. Originally, this cell line was obtained from the rat adrenal pheochromocytoma^33^, and maintained as undifferentiated aggregates. Upon differentiation with NGF, the cells exhibit phenotypes of both acetylcholine and dopaminergic neurons^18^. These cells were also shown to express dopamine transporters (DAT)^32^. Under our standard experimental conditions, NGF-stimulated PC12 cells showed extensive differentiation on collagen coated plates (Fig. 1B) compared to the un-stimulated cells (Fig. 1A). We confirmed the neuronal phenotype of these cells by staining for neurofilaments (Fig. 2B) compared to un-stimulated cells (Fig. 2A). Furthermore, we found that the differentiated cells released high level of DA (Fig. 2C) compared to the medium blank. Based on these observations (Fig. 2B, and C), our results confirmed earlier report^18^ that differentiated PC12 cells behaved like dopaminergic neurons of mammalian CNS.

Cocaine, a widely abused drug, not only shows psycho-stimulatory effects, but also causes severe damage to various brain cells through several toxic responses. Cocaine concentrations to elicit pharmacological response in human addicts have ranged from nano to micromolar^19^ but under *in vitro* cell culture studies, they extended into milli molar range^20–22,34^. In order to study cocaine effects on structural changes in cells, we employed the concentrations spanning from nano, micro and milli molar range. While the selection of lower concentrations in our study was based on earlier reports^19^, the selection of *in vitro* (higher) concentrations of cocaine was based on the LC_50_ and EC_50_ values found in the literature^21,22,35,36^ and on our own assessments of the minimal concentrations of cocaine needed to induce detectable changes under *in vitro* studies^23,37,38^. The use of high concentrations such as 8.8 mM^39^ or 10 mM^20,21^ or even 13 mM^40^ cocaine under *in vitro* conditions is not atypical. Compared to these high doses, we believe that cocaine concentrations (2–4 mM) in our study were within the realm of experimental research. When tested at pharmacological concentrations, cocaine did not induce significant cell death (Fig. 3A) nor did it damage the neurites (Fig. 3B).

In the CNS, cocaine is known to prevent the reuptake of DA by blocking DA transporters on pre-synaptic neurons^41^. Since this inhibition doesn’t prevent further release of DA from pre-synaptic neurons, its concentration at the synaptic cleft could increase several fold^42,43^. In our study, a dose-dependent increase in DA (Fig. 4A) suggests that cocaine caused an inhibition of DA reuptake, resulting its accumulation in the medium. Previous studies showed that cocaine action in PC12 cells was through DAT^32^ which was further confirmed by a dose-dependent inhibition of dopamine reuptake with cocaine treatment in PC12 cells^44^. Although cocaine treatment up to 1 mM did not change the general mitochondrial activity (Fig. 4B) in cells, it significantly affected the membrane potential (Fig. 4C), an observation consistent with previous reports on the loss of membrane potential with cocaine treatment in cells^21,45^ or C6 astroglia-like cells^46^ or in cells outside of CNS^47^. These observations suggest that mitochondria are the primary targets of cocaine-induced toxicity in various cell-types. While a stable membrane potential is essential for normal functioning of mitochondria, its loss due to cocaine exposure causes the cells to switch to anaerobic respiration where pyruvate is converted to lactate. A dose-dependent increase in lactate production in our study (Fig. 5A) confirms anaerobic conditions in cocaine treated cells. We did not observe any ROS in cells treated up to 1 mM cocaine (data not shown). The reason for this is attributed to high GSH level (Fig. 5B) or other antioxidants^48,49^ in cocaine treated cells. Non detection of ROS and lack of cell death (Fig. 3A) up to 1 mM cocaine imply that the increased GSH level (Fig. 5B) through increased glutathione peroxidase^48^ was sufficiently enough to counteract oxidative damage. Lack of both cell death (Fig. 3A) and damage to neurite outgrowth (Fig. 3B) with cocaine at *in vivo* concentrations in our study suggests that one-time cocaine consumption by humans that yields the pharmacological (lower) doses in the brain may not affect the neuronal viability and outgrowth. However, since addicts consume cocaine several times, it is possible that neuronal viability and outgrowth are affected in the long-run. For instance, post-mortem examination of several long-term cocaine addicts showed loss of dopaminergic neurons in the striatal and mid-brain^14^. Some of the factors associated with neuronal loss could be elevated ROS and lipid peroxidation^50^.

Cocaine did not cause cell death up to 1 mM (Fig. 3A). In order to observe specific changes in the cells, it was necessary to increase the treatment concentrations of cocaine which are within acceptable range for other *in vitro* studies^20–22,34^. Studies conducted in non-neuronal cells such as C6 astroglia-like cells indicated that treatment with cocaine at higher concentrations caused drastic cell death^23,46^. Consistent with these observations, when differentiated PC12 cells were treated with similar cocaine concentrations, we observed a significant decrease in the viability (Fig. 6A). These results corroborated with earlier reports, where cocaine cytotoxicity in PC12 cells was confirmed by different assays^51,52^; however, the extent of cocaine cytotoxicity was not quantified in those reports. In our study, the LC_50_ of cocaine was found to be 2.605 mM at 48 h end point. This value was significantly lower than the LC_50_ in C6 astroglia-like cells for cocaine at a 48 h endpoint study^46^ (3.794 mM). The lower LC_50_ value in PC12 cells may suggest that these cells were more sensitive to cocaine compared to C6 astroglia-like cells.

Cocaine treatment at higher concentrations significantly decreased the neurite length (Fig. 6B) and resulted in the loss of intercellular connections. Similar observations were reported earlier in rat^53^ and neuronal cultures treated with cocaine^54^. It is well known that treatment of cells with drug abuse substances often results in cytoplasmic vacuolation, an indication of cell injury. For instance, our previous studies with C6 astroglia-like cells indicated that cocaine exposure caused extensive vacuolation^23,46^, which were in agreement with reports on different cell-types^55–58^ Interestingly, the phenomena of vacuolation was not observed in differentiated PC12 cells when treated with cocaine in our study.

Compounds that interfere with mitochondrial function usually cause oxidative stress in cells^23^. Since cocaine interacts with mitochondria^46^ and impairs the energy metabolism, its toxicity in cells is often associated through oxidative stress. A dose-dependent increase in ROS level (Fig. 7A) in treated cells not only confirmed cocaine’s interaction with mitochondria but also indicated its role in the loss of cell membrane integrity (Fig. 7B). Cocaine-induced ROS generation was reported earlier in different cell-types or animals^23,48,59,60^. ROS is also generated from rapid auto oxidation of DA released from differentiated PC12 cells^61,62^; similarly, dysfunctional mitochondria also generate ROS in cells^63^. However, since DA in our study was released into culture medium, the extracellular auto oxidation of DA is not the source of ROS generation because it cannot be detected by our ROS method. Therefore, the observed ROS (Fig. 7A) with cocaine treatment at higher concentrations ought to be intracellular only. As mitochondria are the main source of ROS formation^64,65^, the intracellular ROS generation (Fig. 7A) suggests its mitochondrial origin. The release of lactate (Fig. 5A) and loss in membrane potential (Fig. 4C) in our study further supports the dysfunctional state of mitochondria for ROS generation. Similar results of ROS generation were reported in undifferentiated PC12 cells treated with cocaine^66^. Since ROS was the cause of cell death in our study, it is speculated that mitochondria were one of the pharmacological targets recruited at higher cocaine concentrations. Increased ROS with cocaine treatment paralleled with the decrease in GSH amount in cells (Fig. 8A), suggesting that ROS was the main cause of cell death (Fig. 6A). This observation was consistent with previous reports where cocaine treatment increased the oxidative damage through decreased antioxidants in the cells^59^. The increase (Fig. 5B) and decrease (Fig. 8A) of GSH level with low and high concentrations of cocaine respectively show the biphasic effect of cocaine in the cells.

Damaged or dysfunctional neurons could trigger inflammation by other non-neuronal cell types -like microglia and astrocytes in the CNS. So, we next investigated whether damage or death of differentiated PC12 cells due to cocaine treatment triggered the release of NO, one of the indicators of inflammatory response. It was found that there was no significant change in NO level between the control and cocaine treated groups under our experimental conditions (Fig. 8B). Lack of induction of NO in our study with cocaine was consistent with earlier reports on different cell types^67^. Since excessive generation of NO is generally associated with cell cytotoxicity, lack of high NO release with cocaine treatment in our study indicates that NO was not the cause of cocaine-induced cytotoxicity.

## Conclusion

Dysfunctional mitochondria^68^, or changes in neuronal structure^69,70^ or loss in neurite-connections could lead to psychiatric illnesses -such as depression^9^, anger, aggressiveness, and paranoia^10^. The findings of dysfunctional mitochondria (Fig. 4C) and neurite-damage (Fig. 6B,C, 2–4 mM) in our study appear to suggest that these changes could be some of the contributing factors for psychiatric illnesses and altered psychology in cocaine addicts. On the other hand, since unusual glucose oxidation due to dysfunctional mitochondria is a common feature in schizophrenia and Alzheimer’s disease^71^, the dose-dependent release of lactate (Fig. 5A), a product of glucose oxidation due to dysfunctional mitochondria (Fig. 4C) in our study, points out the possibility that cocaine users may be increasingly at risk for these neurodegenerative disorders. Further studies are required to ascertain this claim.

Prenatal cocaine exposure could impede dendrite-axon connections in fetus and greatly impact the developing brain^6^. Based on our results (Figs 4C, 6A,B), it can be speculated that the developing fetus could be at risk for neurite damage and damage to neuronal structures if the mother is using cocaine during pregnancy. This may severely affect the brain of developing fetus^6^. As per estimations, around 0.75 million pregnant women are exposed to cocaine every year^72^. Compounds which could prevent cocaine-induced neurite damage could play a vital role in reducing the cocaine toxicity to neurons.

## Materials and Methods

### Materials

RPMI 1640, fetal bovine serum (FBS), penicillin/streptomycin, amphotericin B, phosphate-buffered saline (PBS), Hank’s balanced salt solution (HBSS), and L-glutamine were purchased from Media Tech (Herndon, VA). Horse serum (HS) was obtained from Invitrogen (Waltham, MA). Cocaine-HCl (Ecgonine methyl ester benzoate, MW: 339.8), crystal violet, 2′,7′-dichlorofluorescin diacetate, L-glutaraldehyde, trypan blue, N-1-napthylethylenediamine dihydrochloride (NED), rhodamine (Rh)123 and EDTA (ethylene diamine tetraacetic acid) were supplied by Sigma Chemical Company (St. Louis, MO). All other routine chemicals were of analytical grade.

### Cell culture maintenance

Rat pheochromocytoma PC12 cell line was obtained from American Type Culture Collection (Rockville, MD) and maintained as a suspension culture in RPMI 1640 medium supplemented with 2 mM L-glutamine, 5% (v/v) FBS, 10% (v/v) HS, 100 U/ml penicillin, 100 μg/ml streptomycin and 0.25 μg/ml amphotericin B. Cells were grown in a humidified atmosphere containing 5% CO_2_ in air at 37 °C in an incubator. For sub-culturing, loosely attached cells in the flask were collected and centrifuged at 304 *g* for 4 min at room temperature. The cell pellet was washed with HBSS under sterile conditions, re-suspended in 1 ml complete medium and passed through 25 gauge needle 4–5 times to obtain singlet cells. An aliquot of it was used for cell counting with 0.4% trypan blue on hemocytometer under a light microscope. Dye stained cells (blue) were counted as dead cells, while dye excluded cells were counted as viable. The actual cell numbers were determined by multiplying diluted times compared with initial cell numbers.

### Differentiation of cells

Lyophilized mouse submaxillary gland derived nerve growth factor (NGF, 97% pure, Chemicon International, Inc., Temecula, CA), was reconstituted with sterile DMEM at 0.1 mg/ml, aliquoted and stored at −70 °C. After cell count, cells were diluted in the complete RPMI 1640 medium containing 2.3% FBS, 2.3% HS, 100 ng/ml NGF, and seeded at a low density (2 × 10^4^ cells/0.3 ml per well) in collagen coated 24-well or 96-well sterile culture plates (BD Labware, Bedford, MA). The cells were incubated for five days with the change of NGF containing complete medium once in every 48 h.

### Morphology

For gross cellular morphological evaluation, the crystal violet stained cells were observed under an inverted phase contrast IX-70 Olympus microscope (Olympus, Ontario, NY) with 20x objective. Photomicrographs were taken by an ocular video-camera system (MD35 Electronic eyepiece, Zhejiang JinCheng Scientific & Technology Co., Ltd, HangZhou, China) using C-Imaging System Software (Compix Inc. Cranberry Township, PA).

### Immunocytochemistry and fluorescence Microscopy

Cells were fixed in 4% paraformaldehyde for 15 min, and subsequently permeabilized in 0.5% triton X -100 prepared in PBS for 15 min. Neurofilament 200 kD was determined using immunocytochemistry in fixed permeabilized cells, after blocking with primary rabbit anti-rat NF 200 kD, conjugated to goat anti-rabbit Alexa Fluor® 488 - and nuclear counterstained with PI. Samples were analyzed photographically using a fluorescent/inverted microscope, CCD camera and data acquisition using ToupTek View (ToupTek Photonics Co, Zhejiang, P.R. China) with a 25x objective.

### Dopamine assay

The media (50 μl) from 5 d post seeded or 48 h of cocaine treated cells (0.075, 0.1, 0.125, 0.25, 0.5 and 1 mM) in collagen coated 96-well plates were transferred into new sterile 96-well plates, mixed with 100 μl 0.1 N perchloric acid (PCA) per well and stored at −70 °C until HPLC analysis were performed. On the day of study, the plates were thawed and vortexed gently. From each well, 100 μl of sample was transferred into HPLC vial that contained 700 μl of mobile phase [75 mM sodium phosphate (monobasic), 1.7 mM 1-octanesulfonic acid, 25 μM Na_2_EDTA, 10% acetonitrile and 0.01% triethylamine, pH adjusted to 3.0 by phosphoric acid]. Dopamine was used as a standard reference (0.5 μg/ml). Dopamine level in the samples was detected by reverse phase HPLC C-18 ODS column (4 mm I.D. 7.6 cm, 3 μm particle size) at an isocratic flow rate of 1 ml/min. The HPLC was equipped with an ESA Model 5200 Coulochem III high-sensitivity analytical electrochemical detector. HPLC analysis was carried out using EZChrom software package by Scientific Software (Pleasanton, CA).

### Cocaine treatments

A known amount of cocaine was dissolved in PBS as 1 M stock just prior to the assays. In order to test cocaine toxicity at lower concentrations (0.001, 0.01, 0.05, 0.1, 0.5 and 1 mM final), several working stocks (0.04, 0.4, 2, 4, 20 and 40 mM) were prepared in PBS; similarly, for studies at higher cocaine concentrations (2, 3 and 4 mM final), different working stocks (80, 120 and160 mM) were prepared in PBS. Cocaine samples were always added in a minimum volume of 5 μl per well to prevent pH alterations in culture medium^46^. Final volume in each well was 200 μl. Cells with medium alone or equal volume of vehicle (PBS) in medium served as controls. Cocaine treatments were continued for 48 h at 37 °C, 5% CO_2_ in the incubator without further renewal of medium with the plates capped in normal fashion.

### Evaluation of cell viability

Cytotoxicity of cocaine was evaluated by the dye uptake assay using crystal violet as described previously^73^. Absorbance was measured at 540 nm in a microtiter culture plate reader (Bio-Tek Instruments Inc, Wincoski, VT). The average absorbance values of controls were taken as 100% cell viability. From the treated and control absorbance values, percent cells killed were determined by the following equation: [1− (T/C)] × 100, where T is average absorbance values of treated cells, and C is average absorbance values of control cells.

### Assessment of neurite outgrowth

At the end of 48 h incubation, 400 μl of 0.25% glutaraldehyde was added per well and incubated for 30 min to fix the cells to culture plates. The plates were gently washed three times, and air dried. Then, the cells were stained with crystal violet as described previously^74^. The cells were observed under an inverted phase contrast IX-70 Olympus microscope with 20x objective. Neuronal length, defined as the cellular extensions greater than 2-body diameter in length^24^, was measured at four randomly chosen field areas of total 100 cells per well.

### General mitochondrial metabolic activity

It was performed as per earlier reports^75,76^. The cells in complete media without phenol-red were treated with cocaine at 0.075, 0.1, 0.125, 0.25, 0.5 and 1 mM for 48 h in collagen coated 96-well microtiter plates. One hour prior to the end point, 10 μl of 3-(4,5-dimethylthiazol-2-yl)-5(3-carboxymethonyphenol)-2-(4-sulfophenyl)-2H-tetrazolium (MTS, Promega, Madison, WI) was added per well. Absorbance was taken in a micro plate reader at 490 nm.

### Mitochondrial membrane potential

Cells in collagen coated 96-well plates were treated with cocaine at 0.075, 0.1, 0.125, 0.25, 0.5 and 1 mM for 48 h. At the end of incubation, cells were stained with laser dye fluorescent probe rhodamine (Rh) 123 (10 μM) for 30 min at 37 °C. After discarding excess dye, the cells were washed, air dried and 100 μl PBS was added per well. The plates were read with the excitation filter set at 485 nm and the emission filter at 530 nm in an automatic reader.

### Lactate assay

In order to minimize the serum interaction with lactate reagent (Trinity Biotech, Jamestown, NY), the differentiated cells in collagen coated 96-well plates were treated with cocaine at different concentrations (0.25, 0.5, 1 mM) in reduced serum (0.1% FBS) for 48 h. Lactate was determined by calorimetric method. The content in lactate reagent was dissolved in chromogenic solution consisting of 5.3 mM vanillic acid, 2.9 mM 4-amino antipyrine, and about 4 units of horseradish peroxidase. At the end of treatment period, lactate reagent (20 μl per 200 μl/well) was added, and the plates were incubated at 37 °C until the color was developed (5–10 min). Absorbance was measured at 490 nm in a microtiter culture plate reader.

### GSH level

After treating the differentiated cells with cocaine at lower concentrations (0.075, 0.1, 0.125, 0.25, 0.5, 1 mM) or higher concentrations (2, 3 and 4 mM) for 48 h in collagen coated 96-well microtiter plates, the cells were fixed with 0.25% glutaraldehyde for 30 min, followed by three gentle washings and air drying. Total cellular GSH was assayed as per earlier study^77^. The absorbance was measured at 412 nm in a micro plate reader.

### Intracellular reactive oxygen species (ROS)

Cells were seeded on collagen coated 96-well plates in phenol red free medium containing 0.5% each of FBS and HS. Following incubation of cells for 5 d at 37 °C, 5% CO_2_ in the incubator to facilitate differentiation, cells were treated with cocaine at 2, 3 and 4 mM for 48 h. For measuring the ROS, the cells were stained with 2′,7′ –dichlorofluorescin diacetate (DCFDA, 10 μM final, Sigma Company, St. Louis, MO) for 30 min. After gentle washing and air drying the cells, PBS (100 μl/well) was added. The plates were read with the excitation filter set at 485 nm and the emission filter at 530 nm in an automatic reader (Synergy HTX multimode micro plate reader, BioTek Instruments, Winooski, VT).

### Membrane integrity assay

Cell membrane integrity was determined by measuring LDH release from cells with CytoTox 96 non-radioactive assay kit (Promega) as per kit instructions provided by the manufacturer. In brief, the cells in collagen coated 96-well plates containing 0.5% each of FBS and HS in phenol red free medium were treated with various concentrations of cocaine (2, 3 and 4 mM) for 48 h. Then the media (50 μl) was transferred into new 96-well plates and mixed with equal volume of assay substrate. After 30 min incubation, the color intensity was measured using an automatic micro plate reader at 490 nm.

### Nitric oxide assay

Nitric oxide production with cocaine treatment (2, 3 and 4 mM) in collagen coated 24-well plates was assessed according to the method reported previously^78^ in the complete medium lacking phenol red. In brief, at the end of 48 h treatment with cocaine, 100 µl of medium was transferred into a new 96-titer plate and mixed with an equal volume of Griess reagent (1% sulfanilamide/0.1% NED in 5% phosphoric acid) followed by 10 min incubation in the dark. The absorbance readings at 540 nm were measured in a microtiter culture plate reader.

### Statistical analysis

All results were presented as mean ± standard error mean (SEM). The data were analyzed for significance by one-way ANOVA and then compared by Dunnett’s multiple comparison tests using GraphPad Prism Software, version 3.00 (GraphPad Software, Inc., San Diego, CA). The test value of P < 0.05 and 0.01 were considered significant and highly significant, respectively. The LC_50_ value representing the milli molar concentration of cocaine needed to show 50% response was determined from the graphs as per the method described earlier^79^.

### Data availability

The research data obtained and analyzed in this study are available from the corresponding author on request.
